
JOURNAL INFORMATION
==============================
NLM Title Abbreviation: ScientificWorldJournal
Journal ID: ScientificWorldJournal
NLM Journal ID: 101131163
Journal ID: TSWJ

The Scientific World Journal

ISSN: 2356-6140
EISSN: 1537-744X
Publisher: Wiley

ARTICLE INFORMATION
==============================
PMCID: PMC5917401
PMID: 16633701
DOI: 10.1100/tsw.2006.96
Article ID: 958793
Article version: 1
Subjects: Directions in Science

The Role of Glycosylation in the Secretion of Proteoglycans

Seo Neung-Seon 1 *
McQuillan David J. 2
Höök Magnus 1
1 Center for Extracellular Matrix Biology, Institute of Biosciences and Technology, Texas A&M University Health Science Center, Houston, TX 77030, USA
2 LifeCell Corporation, 1 Millennium Way, Branchburg, NJ 08876, USA
*Neung-Seon Seo: nsseo@ibt.tamhsc.eduAcademic Editor: Martin Goette

Publication date: 2006
Electronic publication date: 2006 Apr 18
Volume: 6
First page: 491
Last page: 493
Received 2006 Mar 1; Accepted 2006 Apr 6
Copyright: Copyright © 2006 Neung-Seon Seo et al.
Copyright year: 2006
License: This is an open access article distributed under the Creative Commons Attribution License, which permits unrestricted use, distribution, and reproduction in any medium, provided the original work is properly cited.
License URL: https://creativecommons.org/licenses/by/3.0/

Keywords: proteoglycans, glycosaminoglycan, decorin, sorting, chaperone, quality control, secretion

==============================
Directions in Science
*TheScientificWorldJOURNAL* (2006) 6, 491–493
ISSN 1537-744X; DOI 10.1100/tsw.2006.96

### The Role of Glycosylation in the Secretion of Proteoglycans

Neung-Seon Seo*[1], David J. McQuillan[2], and Magnus Höök[1]

[1]*Center for Extracellular Matrix Biology, Institute of Biosciences and Technology, Texas A&M University Health Science Center, Houston, TX 77030;* [2]*LifeCell Corporation, 1 Millennium Way, Branchburg, NJ 08876*

**E-mail**: nsseo@ibt.tamhsc.edu; DMcQuillan@lifecell.com; mhook@ibt.tamhsc.edu

*Received March 1, 2006; Accepted April 6, 2006; Published April 18, 2006*

**KEYWORDS**: proteoglycans, glycosaminoglycan, decorin, sorting, chaperone, quality control, secretion

Proteoglycans consist of a protein core and one or more covalently attached glycosaminoglycan (GAG) chains. Proteoglycans are present in intracellular secretion granules, in the extracellular matrix, and on the cell surface. This complexity and wide distribution of proteoglycans reflect the involvement of proteoglycans in a variety of biological processes including tissue growth and repair, cell and tissue morphogenesis and differentiation. In addition proteoglycans contribute to the biophysical properties of a variety of tissues such as skin, bone, and cartilage[1,2].

The GAG chains, including chondroitin sulfate/dermatan sulfate and heparin/heparan sulfate, share a common linkage region, xylose-galactose-galactose-glucuronic acid attached to selected serine residues in the core protein. The formation of this linkage tetrasaccharide is mediated by a discrete set of glycosyltransferases that appear to be common for both types of GAGs. Thus, mutant Chinese Hamster Ovary (CHO) cell lines deficient in xylosyltransferase activity[3], galactosyltransferase I[4], or glucuronosyltransferase I[5] were shown to be unable to synthesize both chondroitin sulfate and heparan sulfate chains that are produced by wild type cells.

Several lines of indirect evidence have suggested that the initial xylosyl transfer represents the committing step in proteoglycan biosynthesis[6,7]. Earlier *in vitro* studies combined with literature surveys identified a putative consensus sequence required for xylosylation of core proteins that comprises a minimum of Ser-Gly-Xxx-Gly[8,9,10]. However, many proteins that contain putative GAG initiation sites escape xylosylation and are secreted as simpler glycoproteins, suggesting that there are requirements beyond the primary amino acid sequence in the vicinity of the xylosylation site that must be met before xylosyl transfer occurs. Thus, the substrate requirements for effective xylosylation are complex. Using a panel of biochemical approaches, we recently confirmed that xylosylation is a critical rate-limiting step in GAG biosynthesis and showed that the conformational information contained in the core protein is important for effective xylosylation[11].

Many eukaryotic cell surface and secreted proteins are post-translationally processed to incorporate N-linked and O-linked carbohydrates[12]. Glycosylation has been shown to play a role in a number of biological processes such as protein folding, stability, targeting, and clearance, as well as cell-matrix and cell-cell interactions[13]. Also, GAGs may contain information for the sorting and secretion of the modified proteins. Studies with canine renal tubule (MDCK) cells have shown that polarized MDCK cells

*Corresponding author.
©2006 with author.
Published by TheScientificWorld, Ltd.; www.thescientificworld.com secreted chondroitin sulfate proteoglycans into the apical medium, while most of heparan sulfate proteoglycans were found in the basolateral medium[14,15]. This observation may suggest that the GAG chains contain structural determinants for sorting and/or that glycosyltransferases involved in the GAG biosynthesis may have a quality control function on proteoglycan secretion. The linkage tetrasaccharide described above serves as a primer for the formation of both chondroitin sulfate and heparan sulfate chains, which is initiated by the attachment of either N-acetyl D-galactosamine or N-acetyl D-glucosamine, respectively[16,17]. The uses in a protein that determine if this is to become a heparan sulfate or a chondroitin sulfate proteoglycan have not been identified, but it is possible that signals that destine the protein for apical or basolateral secretion in a polarized cell also influence the nature of the GAG chain substitution. Structures in the core protein may also affect sorting of the proteoglycan. For example, studies from different groups have shown that a globular domain, G3 in the core protein of aggrecan, affects GAG modification and product secretion[18,19,20,21].

We have investigated the role of glycosylation in decorin secretion using inhibitors of glycosylation and site-specific deletion mutagenesis combined with overexpression of decorin and decorin mutants in CHO cell lines deficient in specific glycosylation steps. Decorin can be efficiently secreted provided that the core protein is substituted with at least one N-linked oligosaccharide or with at least one chondroitin sulfate chain. However, there is severely impaired secretion of core protein devoid of any glycosylation. A decorin core protein mutant devoid of N-linked oligosaccharide attachment sites will not be secreted by CHO cells deficient in xylosyltransferase, or by parental CHO wild type cells if the xylosyltransferase recognition sequence is disrupted[11]. These data suggest that quality control mechanisms sensitive to the absence of N-linked oligosaccharides can be abrogated by interaction of the core protein with the GAG synthetic machinery. We proposed a model of regulation of decorin secretion that has several components, including appropriate substitution with N-linked oligosaccharides and factors involved in GAG synthesis.

To date, there is no direct evidence that any of the GAG and glycosyltransferases involved in GAG synthesis have chaperone or quality control function. The mechanism by which addition of a GAG chain is able to rescue an N-linked oligosaccharide-deficient core protein is unknown. An understanding of the molecule basis of this activity will no doubt provide insight into the role of the GAG chain and glycosyltransferases in GAG biosynthesis.

### ACKNOWLEDGMENTS

We thank Lida Keene for critical reading of the manuscript.

### REFERENCES

1. Hocking, A.M., Shinomura, T., and McQuillan, D.J. (1998) *Matrix Biol.* **17**, 1–19.
2. Iozzo, R.V. (1998) *Annu. Rev. Biochem.* **67**, 609–652.
3. Esko, J.D., Stewart, T.E., and Taylor, W.H. (1985) *Proc. Natl. Acad. Sci. U. S. A.* **82**, 3197–3201.
4. Esko, J.D., Weinke, J.L., Taylor, W.H., Ekborg, G., Roden, L., Anantharamaiah, G., and Gawish, A. (1987) *J. Biol. Chem.* **262**, 12189–12195.
5. Bai, X., Wei, G., Sinha, A., and Esko, J.D. (1999) *J. Biol. Chem.* **274**, 13017–13024.
6. Schwartz, N.B., Roden, L., and Dorfman, A. (1974) *Biochem. Biophys. Res. Commun.* **56**, 717–724.
7. Schwartz, N.B. and Roden, L. (1975) *J. Biol. Chem.* **250**, 5200–5207.
8. Mann, D.M., Yamaguchi, Y., Bourdon, M.A., and Ruoslahti, E. (1990) *J. Biol. Chem.* **265**, 5317–5323.
9. Zhang, L., David, G., and Esko, J.D. (1995) *J. Biol. Chem.* **270**, 27127–27135.
10. Esko, J.D. and Zhang, L. (1996) *Curr. Opin. Struct. Biol.* **6**, 663–670.
11. Seo, N.S., Hocking, A.M., Hook, M., and McQuillan, D.J. (2005) *J. Biol. Chem.* **280**, 42774–42784.
12. Kornfeld, R. and Kornfeld, S. (1985) *Annu. Rev. Biochem.* **54**, 631–664.
13. Varki, A. (1993) *Glycobiology* **3**, 97–130.
14. Kolset, S.O., Vuong, T.T., and Prydz, K. (1999) *J. Cell Sci*. **112(Pt 11)**, 1797–1801.
15. Prydz, K. and Dalen, K.T. (2000) *J. Cell Sci.* **113(Pt 2)**, 193–205.

16. Rohrmann, K., Niemann, R., and Buddecke, E. (1985) *Eur. J. Biochem.* **148**, 463–469.
17. Fritz, T.A., Lugemwa, F.N., Sarkar, A.K., and Esko, J.D. (1994) *J. Biol. Chem.* **269**, 300–307.
18. Kiani, C., Lee, V., Cao, L., Chen, L., Wu, Y., Zhang, Y., Adams, M.E., and Yang, B.B. (2001) *Biochem. J.* **354**, 199–207.
19. Day, J.M., Murdoch, A.D., and Hardingham, T.E. (1999) *J. Biol. Chem.* **274**, 38107–38111.
20. Domowicz, M.S., Pirok, E.W., 3rd, Novak, T.E., and Schwartz, N.B. (2000) *J. Biol. Chem.* **275**, 35098–35105.
21. Luo, W., Kuwada, T.S., Chandrasekaran, L., Zheng, J., and Tanzer, M.L. (1996) *J. Biol. Chem.* **271**, 16447–16450.

**This article should be cited as follows:**

Seo, N.-S., McQuillan, D.J., and Höök, M. (2006) The role of glycosylation in the secretion of proteoglycans. *TheScientificWorldJOURNAL* **6**, 491–493. DOI 10.1100/tsw.2006.96.
